# *Abiotrophia defectiva *knee prosthesis infection: A case report

**DOI:** 10.1186/1752-1947-5-438

**Published:** 2011-09-06

**Authors:** Nadim Cassir, Jean-Charles Grillo, Jean-Noël Argenson, Michel Drancourt, Pierre-Yves Levy

**Affiliations:** 1 Pôle des Maladies Infectieuses et Tropicales, Assistance Publique - Hôpitaux de Marseille et Unité de Recherche sur les Maladies Infectieuses et Tropicales Emergentes, UMR 6236, IRD 198, IFR48, Université de la Méditerranée, Marseille, France; 2 Service de Chirurgie Orthopédique, Hôpital Sainte-Marguerite, Assistance Publique - Hôpitaux de Marseille, France

## Abstract

**Background:**

*Abiotrophia *species have rarely been implicated in osteoarticular infections. We report one case of an *A. defectiva *knee prosthesis infection.

**Case presentation:**

A 71-year-old man of Italian origin presented with pain and swelling of the knee four years after the implantation of a total knee replacement prosthesis. While standard culturing of the synovial fluid resulted in no isolation of microorganisms, the direct inoculation of the synovial fluid into a rich culture medium resulted in the identification of *A. defectiva *by polymerase chain reaction sequencing. Repeated attempts of culturing microorganisms from blood were negative, and echocardiograms and colonoscopies were unremarkable. High-dose amoxicillin for nine months and a two-stage replacement of the knee prosthesis led to full patient recovery by the time of the 12-month follow-up examination.

**Conclusions:**

Because *Abiotrophia *spp. are fastidious microorganisms, it is likely that cases of *Abiotrophia *orthopedic infection are misdiagnosed as culture-negative infections. Direct inoculation of synovial fluids into rich broth medium and further polymerase chain reaction-based detection of culture-negative synovial fluids are key tests for accurate documentation and detection of these infections.

## Background

*Abiotrophia *organisms are nutritionally variant streptococci that form part of the commensal flora of the oral and intestinal mucosae [1]. The detection and identification of *Abiotrophia *organisms may be hampered by their inability to grow in standard media and their microscopic pleomorphism [1]. *Abiotrophia *organisms have been grouped into a unique genus separated from the *Streptococcus *genus based on genetic and phylogenetic analyses, which revealed a low relatedness to other *Streptococcus *organisms [2,3]. *Abiotrophia *organisms cause bacteremia and an estimated 4-6% of all cases of streptococcal endocarditis [2,4]. However, these organisms have rarely been implicated as the causative agents of osteoarticular infections. Indeed, only four cases of orthopedic infections caused by *A. defectiva *have been published. These cases include two instances of septic arthritis, one case of discitis and sacroiliitis and one case of total knee arthroplasty infection [5-8] (Table 1). Here, we report on a fifth case of *A. defectiva *osteoarticular infection through the isolation of the organism and the direct detection of *A. defectiva-*specific DNA sequences through polymerase chain reaction (PCR) detection in the synovial fluid.

**Table 1:** Cases of orthopedic infection caused by *A. defectiva*

Patient	Age/Sex	Dental procedure	Location	Microbiological diagnosis	Endocarditis	Treatment	Outcome
**Patient 1 **[7]	65/F		4-year-old total knee arthroplasty	16S rDNA PCR	No	Cefazolin i.v. 10 days, ciprofloxacin orally 26 days	Relapse four months later: two-stage revision arthroplasty, flucloxacillin orally

**Patient 2 **[5]	90/M	two months before	Knee	Synovial fluid cultures, blood cultures	No	Ceftriaxone i.v., levofloxacin orally six weeks	Improved

**Patient 3 **[6]	75/M	one day before	Knee	Synovial fluid culture, blood cultures	No	Penicillin G i.v. and gentamicin i.v. three weeks, Penicillin G i.v. five weeks, levofloxacin orally four weeks	Improved

**Patient 4 **[8]	51/M	six weeks before	Discitis and sacroiliitis	Blood cultures	Yes	Amoxicillin i.v., gentamicin i.v. and rifampicin orally three weeks, amoxicillin and rifampicin orally 11 weeks	No relapse (12-month follow-up)

**Patient 5 [this case report]**	71/M	one week before	2-year-old total knee arthroplasty	16S rDNA PCR, synovial fluid (blood culture bottles)	No	Two-stage revision arthroplasty. Amoxicillin orally nine months	No relapse (12- month follow-up)

F: female; i.v.: intravenously; M: male

## Case presentation

A 71-year-old man of Italian origin was admitted to our infectious orthopedic department presenting with chronic (two-year) left knee pain, swelling and decreasing ambulation. He had received a total knee prosthesis for left knee osteoarthritis four years prior. The prosthesis had been replaced twice for radiologically confirmed loosening, with the latest replacement being two years prior to admittance. Cultures of synovial fluid collected during both replacements remained sterile after inoculation of standard solid media. Our patient underwent a dental treatment without antibiotic prophylaxis three weeks before the consultation. Upon physical examination, our patient was found to be apyretic. His left knee was swollen and radiating heat, and a moderately sized effusion was present. His white blood cell count indicated 3.82 × 10^3 ^polymorphonuclear cells/mL, his C-reactive protein (CRP) level was 32 mg/L and the erythrocyte sedimentation rate (ESR) was 66 mm (first hour). Our patient was referred to the orthopedic department for sample biopsy and arthroscopic lavage. The synovial fluid was directly inoculated into a set of Bactec Plus Aerobic/F and Bactec Lytic/10 Anaerobic/F bottles (BD Diagnostic Systems, Sparks, MD, USA) and collected in parallel into a sterile tube as part of an "arthritis kit", as previously described [9]. Microscopic examination of the synovial fluid indicated 32 × 10^3 ^white blood cells/mL (98% neutrophil polymorphonuclear cells and 2% monocytes) and 4.77 × 10^3 ^red blood cells/mL without crystals. No microorganisms were observed. Inoculation of the synovial fluid onto 5% sheep-blood agar showed no growth after a five-day incubation period at 37°C under a 5% carbon dioxide (CO_2_) atmosphere. However, the aerobic broth bottle produced Gram-positive cocci after a five-day incubation period at 37°C under continuous automated monitoring for bacterial growth in the medium. Subculturing these organisms on 5% sheep-blood agar and chocolate agar (BioMérieux, Marcy l'Etoile, France) at 37°C under a 5% CO_2 _atmosphere produced colonies. These colonies were identified as *A. defectiva *by 16S rDNA sequencing [10] (99.9% sequence similarity with the GenBank accession number AY879308); E test testing confirmed the isolate to be broadly susceptible with minimum inhibitory concentration of 0.01 mg/L for penicillin G and rifampicin, 0.016 mg/L for amoxicillin, ceftriaxone and vancomycin, 0.02 mg/L for imipenem, 0.1 mg/L for erythromycin and 0.2 mg/L for doxycycline [11]. PCR-based detection of bacterial DNA using universal 16S rDNA primers [10] detected *A. defectiva *DNA in the synovial fluid on the basis of sequence identity to the DNA recovered from the colonies. Three blood cultures incubated under an aerobic atmosphere and one blood culture incubated under an anaerobic atmosphere showed no growth after a five-day incubation period. A transthoracic echocardiography revealed no signs of endocarditis and a colonoscopy was unremarkable. The knee prosthesis was removed, and a temporary cement spacer containing 2 g vancomycin and 40 g cement was inserted [12]. Re-implantation of a total knee was delayed because of an intercurrent venal thrombosis without pulmonary embolism. Our patient received 100 mg/kg/day oral amoxicillin for nine months. The knee pain and swelling subsequently resolved, and the ESR decreased to 22 mm. The one-year follow-up examination indicated complete wound healing, with no evidence of a recurrence of infection, a white blood cell count within the normal range, a CRP value of 7.1 mg/L and an ESR of 17 mm.

## Discussion

In our patient, *A. defectiva *knee prosthesis infection was documented by the isolation and culture of the organism from a synovial fluid recovered under sterile procedures and the parallel detection of *A. defectiva *DNA from this specimen. Although only one joint fluid specimen was available for microbiological investigations, no other detection of *A. defectiva *had been made in our laboratory within the eight-week period preceding isolation of the *A. defectiva *strain here reported. Thus, laboratory contamination is unlikely.

The reported *A. defectiva *isolate was recovered after the direct inoculation of the synovial fluid into a culture broth as part of our protocol for the diagnosis of prosthetic joint infection using an "arthritis kit" procedure. The arthritis kit contains one Bactec Plus Aerobic/F and one Bactec Plus Anaerobic/F for culturing bacteria, one sterile tube for culturing mycoplasma, spirochetes and 16S rRNA-based PCR and one sterile tube for culturing and PCR-based detection of virus, in addition to the informed consent form and laboratory forms. The parallel inoculation of this same fluid specimen onto agar media failed to culture the organism. This illustrates the well-known fastidious nature of *A. defectiva *and indicates that the direct inoculation of the synovial fluid into broth is mandatory for the culture-based diagnosis of *A. defectiva *articular infections. The same holds true for the culture-based diagnosis of arthritis caused by *Kingella kingae *in children [13].

In this patient, the *A. defectiva *infection was documented shortly after a dental procedure without antibiotic prophylaxis. However, this dental procedure was most likely not the portal of entry for *A. defectiva*, as our patient had presented clinical signs of knee prosthesis infection long before the dental procedure. A prospective case-control study indicated that dental procedures were not risk factors for orthopedic prosthetic infection [14]. Indeed, antibiotic prophylaxis before dental interventions in patients with knee arthroplasty lacks evidence-based recommendations [15].

Our patient had a history of repeated early prosthesis loosening, a situation evocative of chronic infection of the prosthesis [16,17]. Tentative documentations remained negative, but these diagnoses lacked direct inoculation of the synovial fluid into broth and molecular detection. Therefore, the loosening episodes of the two prostheses may have been due to a chronic *A. defectiva *infection.

The fastidious nature of *A. defectiva *may explain the rarity of *A. defectiva *orthopedic infections, and cases of *Abiotrophia *infection may be misdiagnosed as culture-negative infections. Accordingly, the role of *Abiotrophia *organisms in osteoarticular infections may be underestimated. A review of the literature indicates that only one case of an orthopedic prosthesis *Abiotrophia *infection has been published, and *A. defectiva *joint infection has been reported in only four cases (Table 1).

## Conclusion

In conclusion, this case illustrates the effectiveness of the "arthritis kit" procedure. By incorporating a systematic direct inoculation of the synovial fluid into broth along with a parallel PCR-based detection of fastidious microorganisms, the diagnosis of orthopedic prosthesis infections, such as those caused by *Abiotrophia *spp., can be optimized.

## Consent

Written informed consent was obtained from the patient for publication of this case report. A copy of the written consent is available for review by the Editor-in-Chief of this journal.

## Competing interests

The authors declare that they have no competing interests.

## Authors' contributions

NC took care of the patient and wrote the manuscript. JCG took care of the patient, reviewed and corrected the manuscript. JNA took care of the patient, reviewed and corrected the manuscript. MD took care of the patient and wrote the manuscript. PYL conceived the study and wrote the manuscript. All authors read and approved the final manuscript.
